# Silencing of GM3 synthase suppresses lung metastasis of murine breast cancer cells

**DOI:** 10.1186/bcr1841

**Published:** 2008-01-03

**Authors:** Yuchao Gu, Junhua Zhang, Wenyi Mi, Jing Yang, Feng Han, Xinzhi Lu, Wengong Yu

**Affiliations:** 1 Department of Molecular Biology, School of Medicine and Pharmacy, Ocean University of China, 5 Yushan Road, Qingdao, China

## Abstract

**Background:**

Gangliosides are sialic acid containing glycosphingolipids that are ubiquitously distributed on vertebrate plasma membranes. GM3, a precursor for most of the more complex ganglioside species, is synthesized by GM3 synthase. Although total ganglioside levels are significantly higher in breast tumor tissue than in normal mammary tissue, the roles played by gangliosides in breast cancer formation and metastasis are not clear.

**Methods:**

To investigate the roles of gangliosides in breast tumor development, GM3 synthase was silenced in the highly metastatic 4T1 cells and over-expressed in the non-metastatic 67NR cells. The behavior of breast cancer cells was examined *in vitro *using migration assay, invasion assay, and soft agar assay. Tumor formation and metastasis *in vivo *were examined using a well established mouse mammary tumor model.

**Results:**

GM3 synthase silencing in 4T1 cells significantly inhibited cell migration, invasion and anchorage-independent growth *in vitro*, and lung metastasis *in vivo*. In addition, over-expression of GM3 synthase in nonmetastatic 67NR cells significantly induced cell migration and anchorage-independent growth. Further studies indicated that activation of the phosphoinositide-3 kinase/Akt pathway, and consequently inhibition of nuclear factor of activated T cell (NFAT)1 expression, could be the mechanism underlying the suppression of breast cancer migration/invasion induced by GM3 synthase silencing.

**Conclusion:**

Our findings indicate that GM3 synthase silencing suppressed lung metastasis in murine breast cancer cells. The molecular mechanism that underlies GM3 synthase mediated migration and invasion was inhibition of the phosphoinositide-3 kinase/Akt pathway. The findings suggest that GM3 synthase may be of value as a therapeutic target in breast cancer.

## Introduction

Gangliosides are sialic acid containing glycosphingolipids that are ubiquitously distributed on vertebrate plasma membranes [1]. They participate in the regulation of various cellular functions, including cell proliferation, apoptosis, migration, and invasion [2-4]. Numerous studies have demonstrated that abnormal ganglioside expression is strongly associated with the malignancy of cancer cells [5]. The ganglioside content is upregulated in some metastasizing tumor cells, such as lymphoma cells, fibrosarcoma cells, and melanoma cells [6-8]. However, there is an inverse relation between metastasis properties and ganglioside content in some types of tumors, for instance bladder tumor and liver carcinoma [4,9,10]. Some observations indicate that the effects of gangliosides on tumor metastasis are dependent on the ganglioside species and/or tumor-type specificity. For example, the effects of a given ganglioside, such as GM3, on metastatic potential differ between tumor cell types [10-12]. On the other hand, the metastatic potential of a given tumor cell type, such as melanoma cells, may be enhanced or inhibited by different gangliosides [3,11,13]. Hence, there is a need to define the involvement and molecular mechanisms of gangliosides in metastasis of each cancer type.

Among the various gangliosides, GM3 contains the simplest ganglioside oligosaccharide and it serves as a precursor for most of the more complex ganglioside species (for example, GD3, GM2, GD2, and so on) [14]. GM3 is synthesized by transfer of sialic acid from cytidine 5'-monophosphate (CMP)-sialic acid to nonreduced terminal galactose residue of lactosylceramide through the α-2,3-glycosyl bond, and the reaction is catalyzed by GM3 synthase (CMP-N-acetylneuraminic acid:lactosylceramide 2,3-sialyltransferase [EC 2.4.99.9]; also known as ST3Gal V, GM3S [the term used in the present report], and Siat9) [15]. Overexpression and suppression of GM3S expression are approaches used to determine the effects of endogenous gangliosides on the metastatic process [2,16].

Total gangliosides in breast tumor tissues and sera from breast cancer patients were significantly higher than in those from healthy individuals [17,18]. GM3 and some other gangliosides (such as GD3 and GM2) have been reported to be potential targets for breast cancer immunotherapy [19-22], chemotherapy [23], and radiotherapy [24]. Although ganglioside levels have been demonstrated to be elevated in breast cancer, there are no reports in the literature regarding the roles played by gangliosides in breast cancer metastasis.

In this study, in order to investigate the roles played by endogenous gangliosides in breast cancer formation and metastasis, GM3S was silenced or over-expressed in different breast tumor cell lines. The effects of GM3S expression on the malignant properties of breast tumor cells and the molecular mechanisms of ganglioside-mediated migration and invasion were evaluated.

## Materials and methods

### Cells and cell cultures

67NR and 4T1 (kindly provided by Dr Fred R Miller at Karmanos Cancer Institute) are mammary tumor cell lines characterized by enhanced lung metastatic potential that were derived from a single, spontaneously arising mouse mammary tumor [25]. The nonmetastatic 67NR cells fail to leave the primary site, whereas the 4T1 cells can metastasize to lung, liver, bone, and brain via the hematogenous route. They were cultured in Dulbecco's modified Eagles medium supplemented with 10% fetal calf serum (DME-10), 1 mmol/l mixed nonessential amino acids, and 2 mmol/l L-glutamine.

### Plasmid construction

The dual promoter small interfering RNA (siRNA) expression vector pMEHMpuro was constructed based on a self-inactivating murine stem cell virus plasmid, namely pMSCVpuro. First, the *Hind*III restriction site of pMSCVpuro was deleted by digesting the plasmid with *Hind*III, blunting the 3' recessed ends and re-ligating. The enhanced green fluorescent protein (EGFP) gene was then inserted into the *Bgl*II/*Xho*I sites of *Hind*III restriction site deleted pMSCVpuro vector; the resulting construct was named pMSCVpuro-EGFP. Then, the dual promoter siRNA expression cassette was constructed as described previously [26] and inserted into the *Xho*I/*Eco*RI-digested pMSCVpuro-EGFP plasmid to form pMEHMpuro. In order to generate siRNA vectors, three pairs of oligonucleotides, corresponding to different regions of murine GM3S encoding gene, were annealed and inserted into *Bgl*II/*Hind*III double digested pMEHMpuro: siGM3S1, 5'-AGC TTA AAA AGT AAG GTT GAA CAG TGC GCC TTT TTA-3' and 5'-GAT CTA AAA AGG CGC ACT GTT CAA CCT TAC TTT TTA-3'; siGM3S2, 5'-AGC TTA AAA AGA CTG CCT TCG ACA TCC TTC TTT TTA-3' and 5'-GAT CTA AAA AGA AGG ATG TCG AAG GCA GTC TTT TTA-3'; and siGM3S3, 5'-AGC TTA AAA AGT GTG ACC ACA GAG ACC AAG TTT TTA-3' and 5'-GAT CTA AAA ACT TGG TCT CTG TGG TCA CAC TTT TTA-3'. This process yielded the vectors pMEHM-siGM3S1 (siGM3S1), pMEHM-siGM3S2 (siGM3S2), and pMEHM-siGM3S3 (siGM3S3), respectively. In addition, annealed oligonucleotides (5'-AGC TTA AAA AGT TCC GTA TGT TGC ATC ACC TTT TTA-3' and 5'-GAT CTA AAA AGG TGA TGC AAC ATA CGG AAC TTT TTA-3') that did not match any known mouse gene was inserted into *Bgl*II/*Hind*III double digested pMEHMpuro, yielding pMEHM-siMock. The full-length GM3S encoding gene was amplified from the total RNA of 4T1 cells using RT-PCR and inserted into *Xho*I/*Eco*RI double digested pMSCVpuro, resulting in the GM3S expression vector pMSCV-GM3S. All of the resulting constructs were confirmed by DNA sequencing.

### DNA transfection and selection

4T1 cells were transfected with various GM3S-targeting RNA interference and mock plasmids. 67NR cells were transfected with GM3S and pMSCVpuro vectors. Transfection was carried out using LipofectAMINE 2000 (Invitrogen, Carlsbad, CA, USA), and the transfected cells were cultured in the medium supplied with 4 μg/ml puromycin for 2 weeks. The obtained cells were named 4T1-siMock, 4T1-siGM3S1, 4T1-siGM3S2, 4T1-siGM3S3, 67NR-Control and 67NR-GM3S.

### RNA isolation and real-time RT-PCR

Total RNA was isolated from cells using Trizol (Invitrogen) and reverse transcription was carried out using the High Capacity cDNA Archive Kit (Applied Biosystems, Foster City, CA, USA). Real-Time PCR was carried out using *Power *SYBR Green Master PCR mix (Applied Biosystems, Warrington, UK) in triplicate, and the reactions were conducted on a 7500 real-time PCR system (Applied Biosystems, Singapore). The relative quantitative analysis was normalized to endogenous control glyceraldehyde-3-phosphate dehydrogenase (GAPDH). The mouse GM3S forward primer was 5'-GCT TCA AGC AAT GGT AAA AAA TGA-3', and its reverse primer was 5'-TTC TGC CAC TTG CTT CCA AA-3'. The mouse phosphatase and tensin homolog (PTEN) forward primer was 5'-TGA AGA CCA TAA CCC ACC ACA-3', and its reverse primer was 5'-TCA TTA CAC CAG TCC GTC CCT-3'. Mouse GAPDH forward primer was 5'-AAT TCA ACG GCA CAG TCA AGG-3', and its reverse primer was 5'-TGT TAG TGG GGT CTC GCT CC-3'.

### Immunoblotting (IB) analysis

Cells were lysed in lysis buffer (50 mmol/l Tris-HCl [pH 7.4], 150 mmol/l NaCl, 1% NP40, 1 mmol/l EDTA, 1 mmol/l Na_3_VO_4_, 10 mmol/l NaF) containing a protease inhibitor cocktail (Roche, Nutley, NJ, USA). Protein samples (50 μg) were separated by 12% SDS-PAGE and transferred to Immobilon-P membranes (Millipore, Bedford, MA, USA). Antibodies to phosphorylated and total Akt (Cell Signaling, Beverly, MA, USA), phosphorylated (Ser380/Thr382/383) and total PTEN (Cell Signaling), NFAT1 (Santa Cruz, CA, USA), and GAPDH (Santa Cruz) were used, with detection by ECL-detecting reagent (Amersham Biosciences, Buckinghamshire, UK). Quantification of the blots was conducted using Image-Pro Plus software (version 6.0; Media Cybernetics, Bethesda, MD, USA).

### Flow cytometry analysis

The flow cytometry procedure was as described previously [27]. Briefly, cells were trypsinized and incubated with anti-GM3 or anti-GD3 monoclonal antibodies (Seikagaku Corporation, Tokyo, Japan) and then labeled with rat anti-mouse IgM-P-RE (Southern Biotechnology Associates, Birmingham, AL, USA). A total of 1 × 10^4 ^labeled cells were analyzed using a cytometer (Becton Dickinson, Franklin Lakes, NJ, USA).

### Cell proliferation assay

Cells were seeded 1 × 10^4 ^per well in a 96-well plate. Cells were allowed to grow for 24 hours. Then, 20 μl of 3-(4,5-dimethylthiazol-2-yl)-2,5-diphenyltetrazolium bromide (MTT) (5 mg/ml) was added to each well. After 4 hours of incubation at 37°C, cells were lysed by addition of 200 μl dimethylsulfoxide. Absorbance was measured at 570 nm using a Rainbow microplate reader (Tecan, Groding/Salzburg, Austria).

### Anchorage-independent cell growth

Cells (5 × 10^3 ^to 1 × 10^4^) were suspended in 1 ml top agar medium (DME-10 supplied with 0.4% agar), in the presence or absence of phosphoinositide-3 kinase (PI3K)-specific inhibitor LY294002 (Merck, Nottingham, UK). The cell suspension was then overlaid onto 1.5 ml bottom agar medium (DME-10 supplied with 0.8% agar) in six-well tissue culture plates in triplicate. DME-10, with or without LY294002, was added to the plates every 3 days as a feeder layer. On day 12, the number of colonies was counted in six random fields at 40× magnification.

### Cell migration and invasion assay

Cell migration was assayed using Transwell chambers (6.5 mm; Corning, New York, USA) with 8 μmol/l pore membranes. The lower chamber was filled with 600 μl NIH-3T3 conditioned medium containing 20 μg/ml fibronectin (BD Biosciences, Bedford, MA, USA) with or without 2 μmol/l LY294002. Cells (5 × 10^4^) were suspended with 100 μl upper medium (Dulbecco's modified Eagles medium with 1% fetal calf serum) and plated into the upper chamber with or without 2 μmol/l LY294002. After 16 hours, the number of cells appearing by crystal violet staining on the undersurface of the polycarbonate membranes was scored visually in five random fields at 100× magnification using a light microscope.

For invasion assays, the upper face of the membrane was covered with 70 μl Matrigel (1 mg/ml; BD Biosciences). The invasion assay procedure was the same as for the migration assay, except that the incubation time of the experiment was prolonged to 24 hours.

### Primary tumor growth and lung metastases assay

These procedures were performed as described previously [25,28] with minor modifications. Female BALB/c mice, aged 8 to 10 weeks, were used in the experiment. In brief, mice (six to eight per group) were anesthetized with sodium pentobarbital (50 mg/kg body weight), and tumor cells (5 × 10^5^) in 10 μl DME-10 were injected into the mammary gland. The weight of the primary tumors and the number of metastatic nodules on the lung surface were evaluated 30 days after the tumor cells injection. The animals were housed and cared for in accordance with the guidelines established by the National Science Council of Republic of China. The lung tissues (six lungs per group) from *in vivo *experiments described above were fixed in 10% neutral buffered formalin and embedded in paraffin, and then thick sections (4.0 μm; three sections per lung tissue) were cut and stained with hematoxylin and eosin.

### Statistic analysis

All of the results were repeated in at least three independent experiments and consistently yielded similar results. Data were analyzed by the Student-Newman-Keuls test using the SPSS 11.0 software program (SPSS Inc., Chicago, IL, USA). *P *< 0.05 was considered statistically significant. Results are expressed as mean ± standard error of the mean.

## Results

### GM3S is silenced or overexpressed in breast cancer cell lines

**Figure 1:**
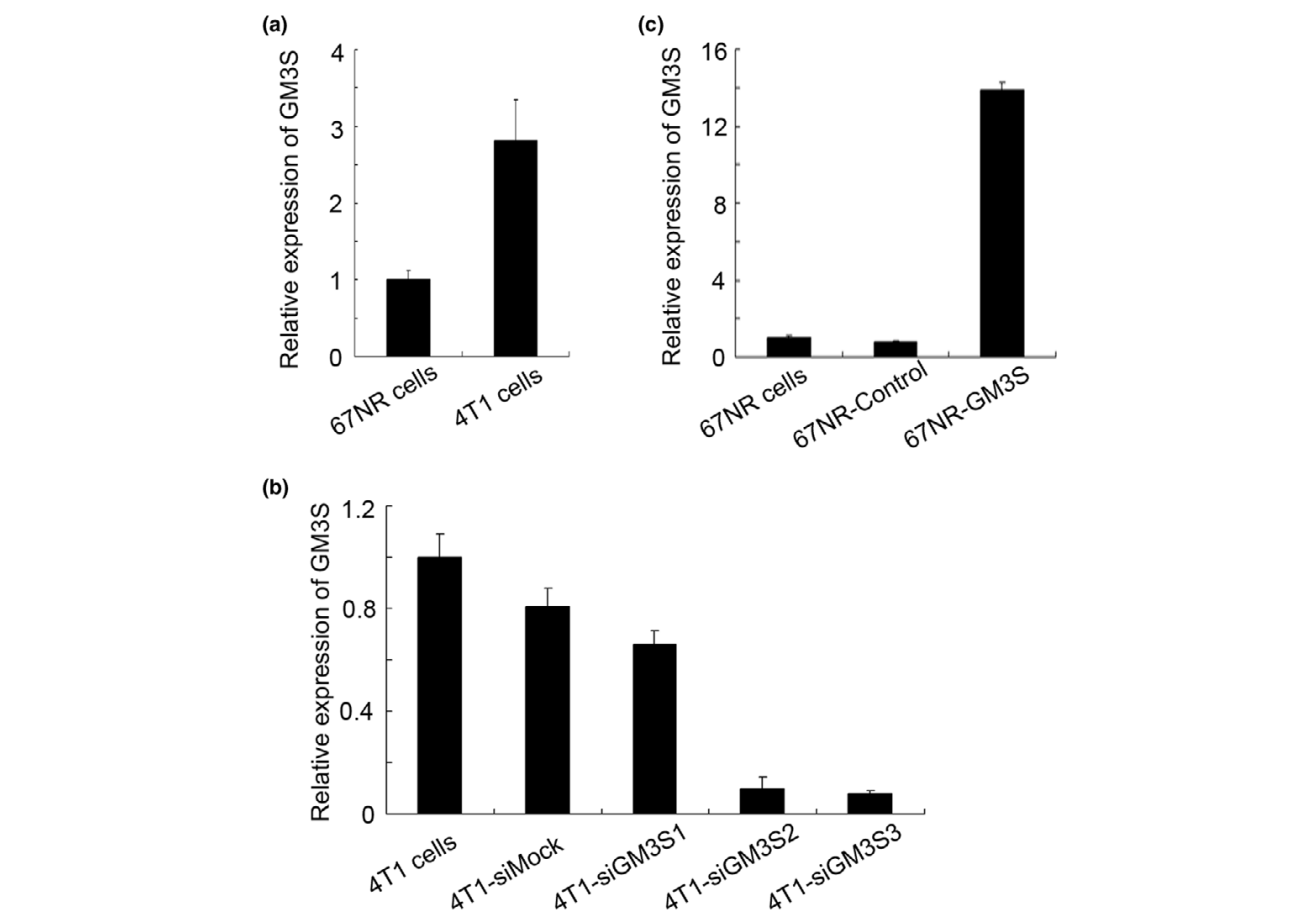
Analysis of GM3S expression of breast tumor cell lines by real-time RT-PCR. **(a) **GM3S mRNA expression was higher in metastatic 4T1 cells than in noninvasive 67NR cells. **(b, c) **Relative GM3S mRNA expression in **(b) **GM3S-silenced 4T1 cells and in **(c) **GM3S over-expressing 67NR cells. CMP, cytidine 5'-monophosphate; GM3S, CMP-N-acetylneuraminic acid:lactosylceramide 2,3-sialyltransferase.

**Figure 2:**
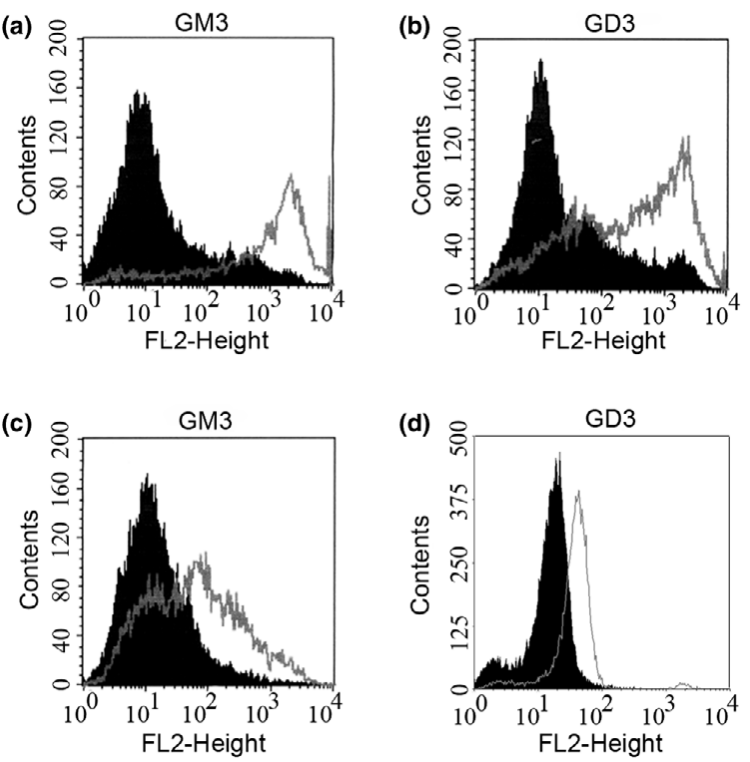
Analysis of ganglioside content on cell surface by flow cytometry. Expression levels of **(a) **GM3 and **(b) **GD3 gangliosides on the surface of GM3S-silenced 4T1 cells using anti-GM3 and anti-GD3 monoclonal antibody (shaded peaks: 4T1-siGM3S2; unshaded peaks: 4T1-siMock). Expression levels of **(c) **GM3 and **(d) **GD3 gangliosides on the surface of GM3S over-expressing 67NR cells using anti-GM3 and anti-GD3 monoclonal antibody (shaded peaks: 67NR-Control; unshaded peaks: 67NR-GM3S). CMP, cytidine 5'-monophosphate; GM3S, CMP-N-acetylneuraminic acid:lactosylceramide 2,3-sialyltransferase.

We found that the expression of GM3S mRNA was greater in the highly metastatic 4T1 tumor cells than in the nonmetastatic 67NR cells (Figure 1a). These observations led us to regard GM3S to be a potential candidate for orchestrating metastasis in breast cancer. To determine whether GM3S played an important role in breast cancer malignancy, expression of GM3S in the highly metastatic 4T1 cell line was silenced by RNA interference. The expression of the GM3S siRNAs siGM3S2 and siGM3S3, but not that of siGM3S1, drastically reduced the expression of GM3S mRNA in 4T1 cells (Figure 1b). The results of flow cytometry analysis indicate that GM3S knockdown significantly suppressed the expression of the ganglioside GM3 (Figure 2a) and the more complex ganglioside GD3 (Figure 2b). On the other hand, GM3S was over-expressed in 67NR cells. The expression of GM3S was upregulated (Figure 1c) and the levels of expression of GM3 (Figure 2c) and GD3 (Figure 2d) gangliosides were enhanced. These GM3S-reconstructed cells enabled us to identify the effects of GM3S expression on the malignant properties of breast cancer cells, and these cell lines also appeared to be useful for analyzing the roles played by gangliosides in breast cancer cells.

### GM3S expression enhances the anchorage-independent growth of breast cancer cells

**Figure 3:**
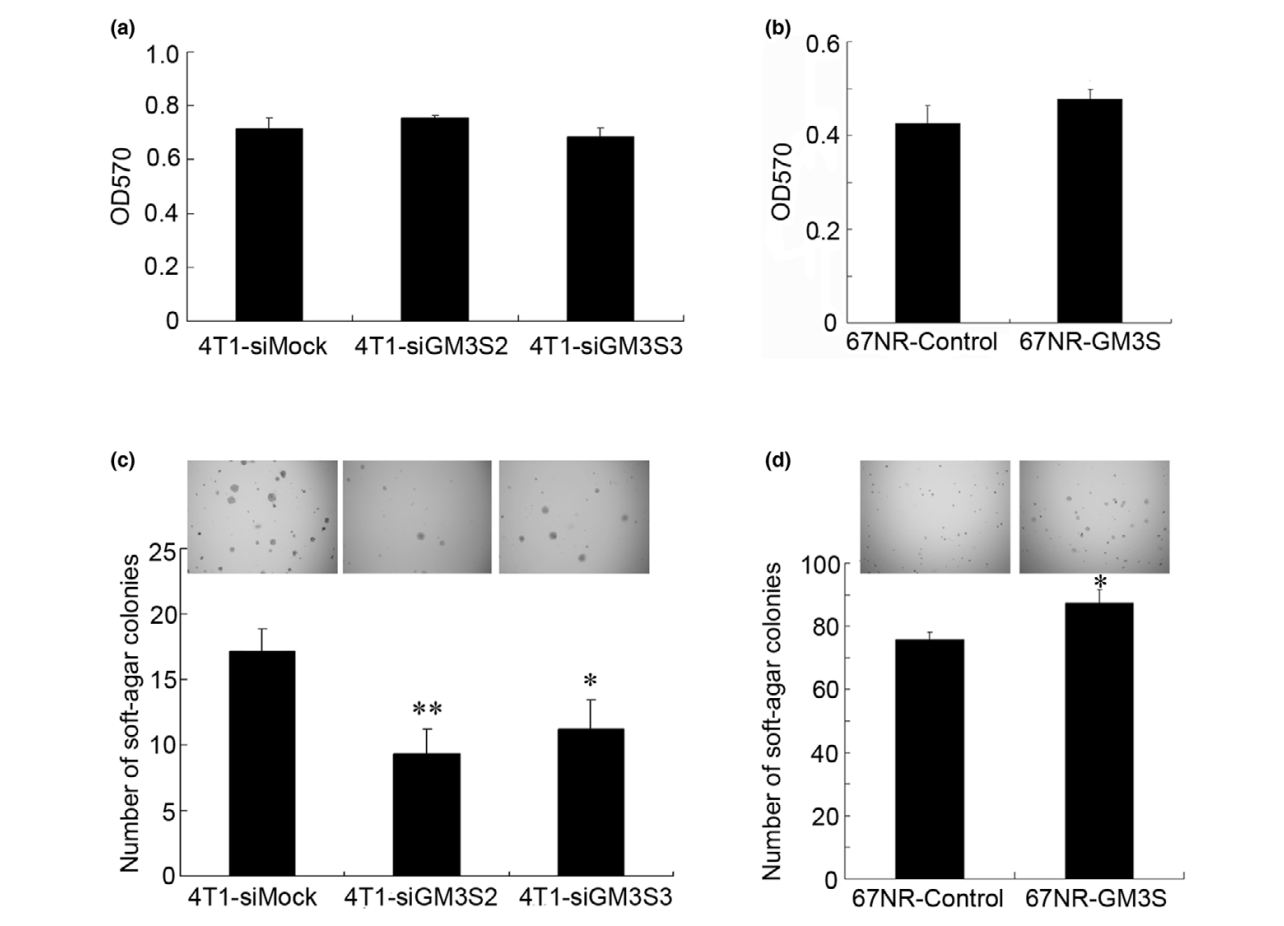
Analysis of cell proliferation and anchorage-independent growth. Proliferation of **(a) **GM3S-silenced 4T1 cells and **(b) **GM3S over-expressing 67NR cells was analyzed by MTT assay. Anchorage-independent growth of **(c) **GM3S-silenced 4T1 cells (5 × 10^3 ^cells/well) and **(d) **GM3S over-expressing 67NR cells were analyzed by soft agar assays (1 × 10^4 ^cells per well). *P < 0.05, **P < 0.01. CMP, cytidine 5'-monophosphate; GM3S, CMP-N-acetylneuraminic acid:lactosylceramide 2,3-sialyltransferase.

To investigate whether GM3S expression affected cell proliferation and anchorage-independent growth, MTT and soft agar colony assays were conducted. Silencing of GM3S in 4T1 cells and over-expression of GM3S in 67NR cells did not affect the proliferation (Figure 3a,b). The results were also verified by cell counting experiments (data not shown). Interestingly, GM3S knockdown in 4T1 cells significantly reduced the number of colonies and slightly decreased the colony size (Figure 3c), whereas 67NR cells over-expressing GM3S formed much larger colonies than did control cells, and the number of colonies was slightly increased (Figure 3d). These results indicated that endogenous GM3S was important for maintaining the anchorage-independent growth of breast tumor cells.

### GM3S expression enhances migration and invasion of breast cancer cells

**Figure 4:**
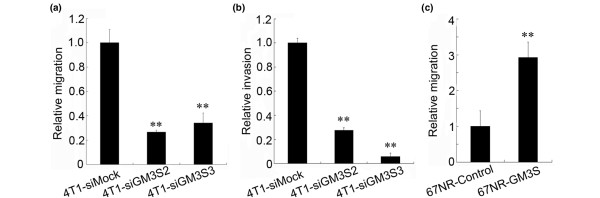
Migration and invasion assays. The relative **(a) **migration and **(b) **invasion assays for GM3S-silenced 4T1 cells. **(c) **The relative migration assays for GM3S over-expressing 67NR cells. **P < 0.01. CMP, cytidine 5'-monophosphate; GM3S, CMP-N-acetylneuraminic acid:lactosylceramide 2,3-sialyltransferase.

To investigate the role played by GM3S in breast cancer cell metastasis, we conducted migration and invasion assays *in vitro*. As shown in Figure 4a and 4b, the migration and invasion abilities were inhibited by silencing of GM3S in 4T1 cells. Over-expression of GM3S in noninvasive 67NR cells enhanced their ability to migrate (Figure 4c). However, over-expression of GM3S was unable to transform noninvasive 67NR cells into invasive ones (data not shown). These findings suggest that GM3S expression is essential but not sufficient for breast cancer cell migration and invasion.

### Inhibition of GM3S expression suppresses lung metastases of breast cancer cells

**Figure 5:**
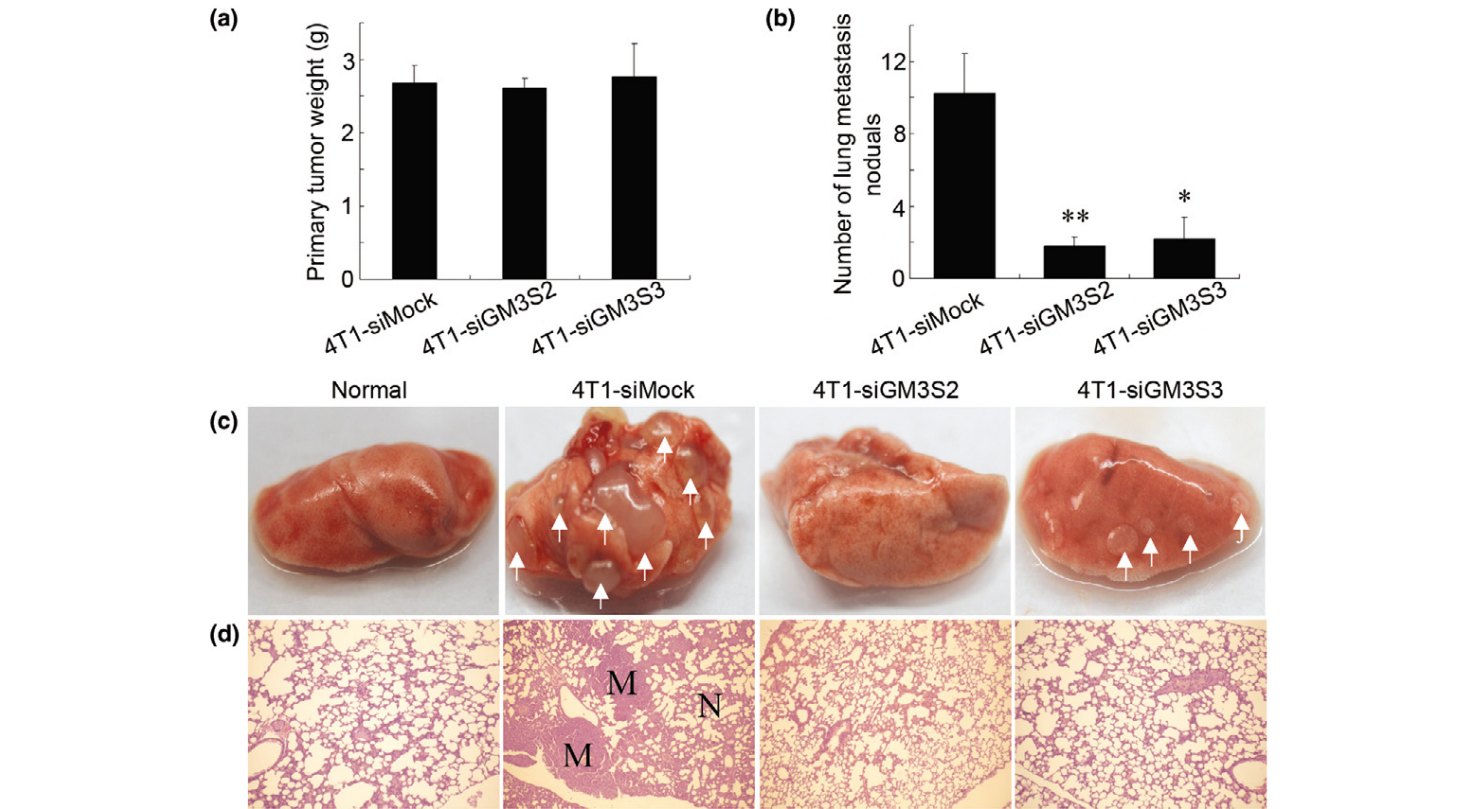
Tumor formation and lung metastases 30 days after tumor implantation. **(a) **Primary tumor weights. **(b) **The average numbers of lung metastatic nodules. **(c) **Representative photos of the lungs. The arrows point to the metastatic nodules in lung. **(d) **Representative hematoxylin and eosin staining sections of the lungs were photographed at 40× magnification. *P < 0.05. M, metastatic nodule; N, normal.

Based on the roles played by GM3S expression in migration/invasion and anchorage-independent growth of breast cancer cells described above, we next examined the effects of GM3S silencing on tumor formation and metastasis of breast cancer cells. Consistent with the results *in vitro*, GM3S silencing in 4T1 cells had no effect on primary tumor weight (Figure 5a) but it dramatically reduced the number of visible metastatic nodules on the lung surface of tumor-bearing mice (Figure 5b,c). Histologic analyses confirmed that the number of micrometastatic lesions was significantly reduced in lungs of mice carrying 4T1-siGM3S2 and 4T1-siGM3S3 tumors (Figure 5d).

### GM3S promotes breast cancer cells migration and invasion through the PI3K/Akt pathway

**Figure 6:**
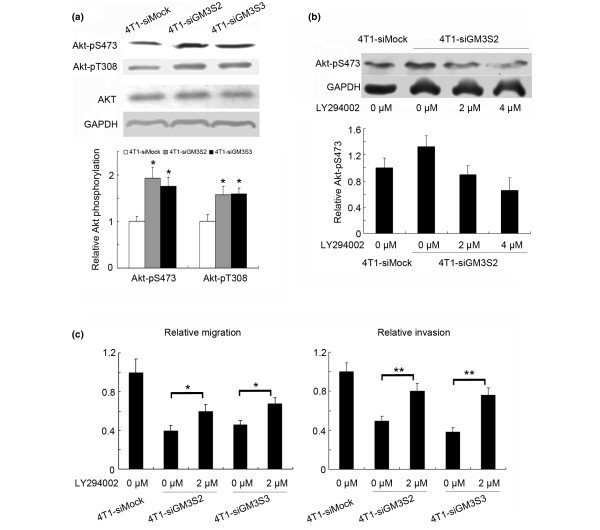
Involvement of the PI3K/Akt in suppression of breast cancer migration/invasion. Activation of the PI3K/Akt pathway is the causative mechanism for the suppression of breast cancer migration/invasion induced by GM3S silencing. **(a) **Upper panels: the expression and activation of Akt detected by immunoblotting with anti-Akt, anti-p-Akt Ser (473), and anti-p-Akt Thr (308) antibodies. Lower panels: quantitation of these blots after normalization with the GAPDH blot. **(b) **Upper panels: PI3K inhibitor LY294002 restored the phosphorylation of Akt in 4T1-siGM3S2 cells. Lower panels: the quantitation of the blots after normalization with the GAPDH blot. **(c) **The migration and invasion abilities of GM3S-silenced 4T1 cells were enhanced by LY294002. *P < 0.05, **P < 0.01. CMP, cytidine 5'-monophosphate; GAPDH, glyceraldehyde-3-phosphate dehydrogenase; GM3S, CMP-N-acetylneuraminic acid:lactosylceramide 2,3-sialyltransferase; PI3K, phosphoinositide-3 kinase.

To investigate the molecular mechanism underlying GM3S-mediated migration and invasion, some metastasis-associated signaling pathways were assayed by immunoblotting. We found that suppression of GM3S significantly enhanced the phosphorylation of Akt at two key residues, namely Thr308 and Ser473 (Figure 6a). Further studies demonstrated that the PI3K specific inhibitor LY294002 (2 μmol/l) restored Akt phosphorylation to the level exhibited by 4T1-siMock cells (Figure 6b) and partially compensated for the inhibition of cell migration/invasion induced by the GM3S knockdown (Figure 6c).

**Figure 7:**
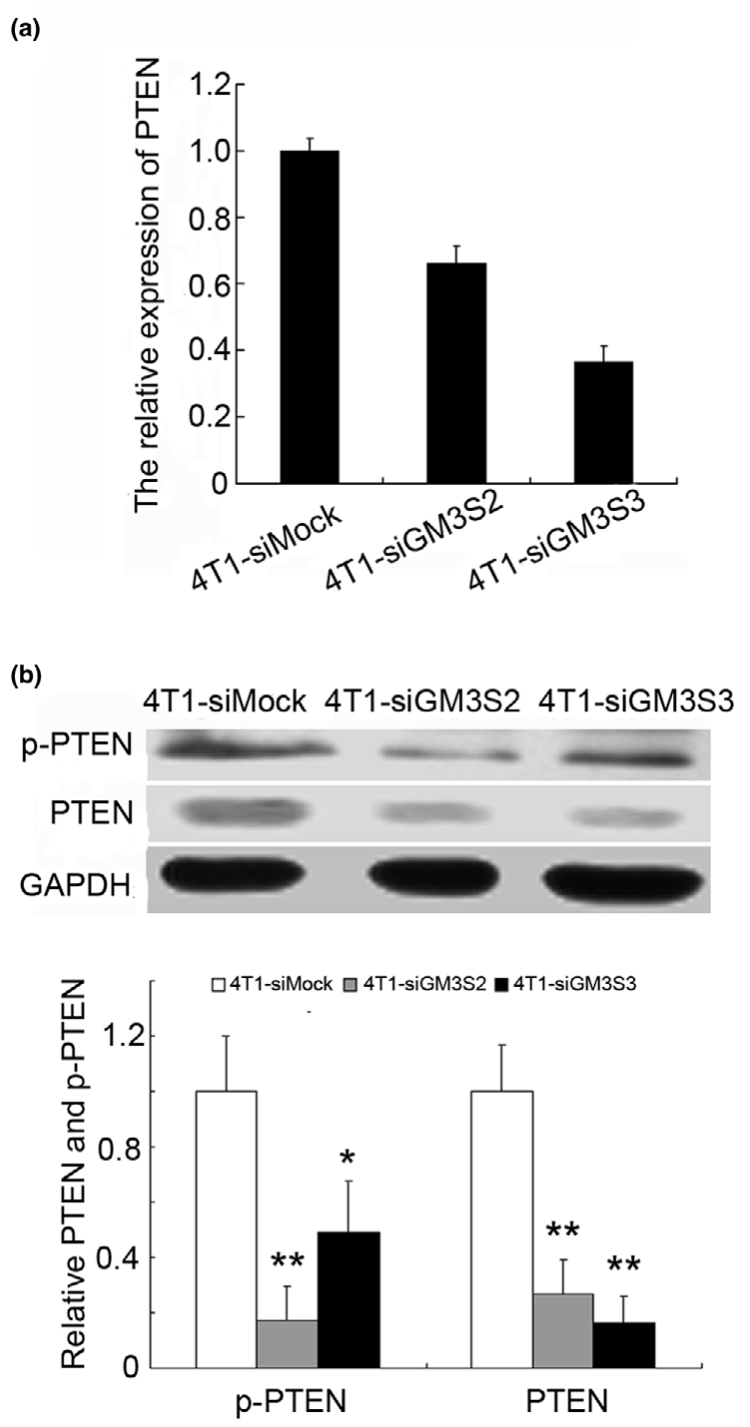
Expression of PTEN was inhibited by GM3S silencing. **(a) **Relative PTEN expression in GM3S-knockdown 4T1 cells analyzed by real-time RT-PCR. **(b) **Upper panels: expression and activation of PTEN detected by immunoblotting. Lower panels: quantitation of these blots after normalization with the GAPDH blot. *P < 0.05, **P < 0.01. CMP, cytidine 5'-monophosphate; GAPDH, glyceraldehyde-3-phosphate dehydrogenase; GM3S, CMP-N-acetylneuraminic acid:lactosylceramide 2,3-sialyltransferase; p-PTEN, phosphor-PTEN Ser380/Thr382/383; PTEN, phosphatase and tensin homolog.

PTEN is a major negative regulator of the PI3K/Akt signaling pathway. In the present study, GM3S inhibition suppressed the expression and phosphorylation of PTEN in 4T1 cells (Figure 7a,b). These findings suggest that the inhibition of PTEN expression at least partially contributed to activation of the PI3K/Akt pathway in 4T1 cells in which GM3S had been knocked down.

**Figure 8:**
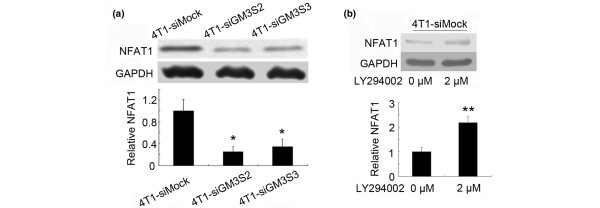
Expression of NFAT1 was inhibited through the PI3K/Akt pathway in GM3S-knockdown 4T1 cells. **(a) **Upper panels: expression of NFAT1 was detected by immunoblotting. Lower panels: quantitation of these blots after normalization with the GAPDH blot. **(b) **Upper panels: expression of NFAT1 was enhanced by the PI3K inhibitor LY294002. Lower panels: quantitation of these blots after normalization with the GAPDH blot. *P < 0.05, **P < 0.01. CMP, cytidine 5'-monophosphate; GAPDH, glyceraldehyde-3-phosphate dehydrogenase; GM3S, CMP-N-acetylneuraminic acid:lactosylceramide 2,3-sialyltransferase; NFAT, nuclear factor of activated T cell; PI3K, phosphoinositide-3 kinase.

**Figure 9:**
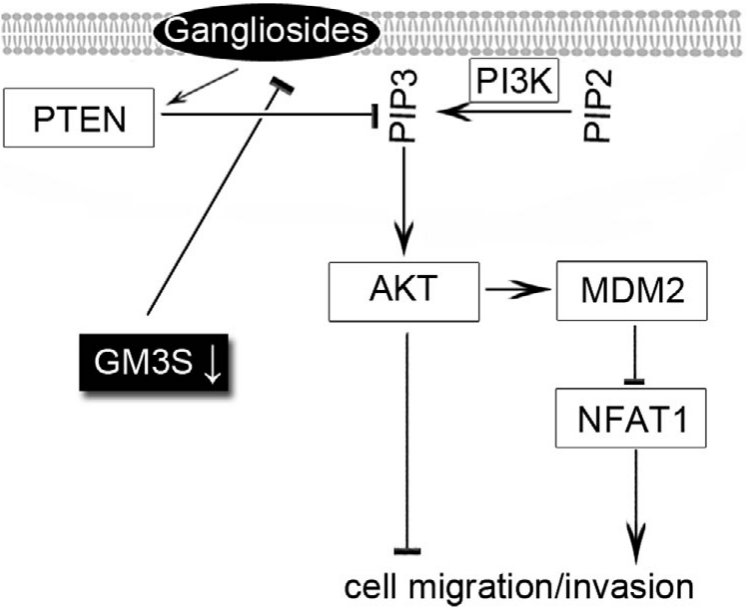
Effects of GM3S on Akt-associated cell migration/invasion in breast cancer cells. This schematic diagram shows that GM3S silencing reduced expression of gangliosides and activated Akt signaling via inhibition of PTEN expression (the major negative regulator of the PI3K/Akt signaling). It also shows that activation of Akt signaling inhibits cell migration and invasion, probably via downregulation of NFAT1. CMP, cytidine 5'-monophosphate; GM3S, CMP-N-acetylneuraminic acid:lactosylceramide 2,3-sialyltransferase; PI3K, phosphoinositide-3 kinase; PTEN, phosphatase and tensin homolog.

We further investigated the downstream effectors of PI3K/Akt pathway. GM3S knockdown inhibited NFAT1 expression (Figure 8a), and inhibition of the PI3K/Akt pathway induced NFAT1 expression (Figure 8b). In conclusion, as illustrated in Figure 9, GM3S knockdown suppressed PTEN expression and resulted in activation of Akt signaling, which reduced the expression of NFAT1 and inhibited cell migration and invasion.

### PI3K/Akt pathway has no effect on anchorage-independent growth of breast tumor cells

**Figure 10:**
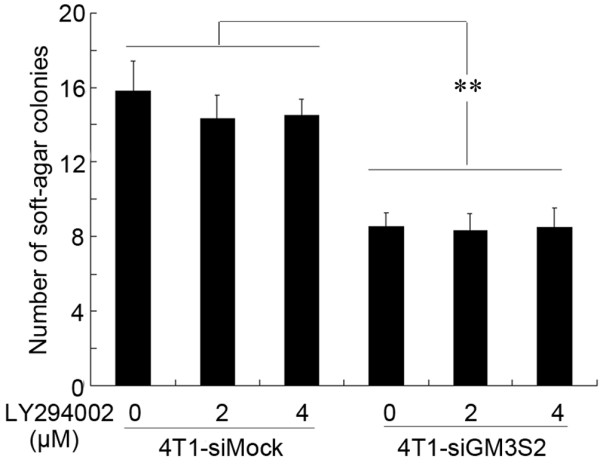
Effects of PI3K/Akt signal on the anchorage-independent growth of breast cancer cells. The anchorage-independent growth of both GM3S-knockdown 4T1 cells and the mock transfected 4T1 cells (5 × 10^3 ^cells/well) were not affected by the inhibition of the PI3K/AKT pathway. CMP, cytidine 5'-monophosphate; GM3S, CMP-N-acetylneuraminic acid:lactosylceramide 2,3-sialyltransferase; PI3K, phosphoinositide-3 kinase.

To test whether activation of the PI3K/Akt pathway was responsible for the anchorage-independent growth affected by GM3S knockdown in 4T1 cells, we inactivated the PI3K/Akt pathway with LY294002. The colony forming abilities of both GM3S-knockdown 4T1 cells and the mock transfected cells were unaffected by inhibition of the PI3K/AKT pathway (Figure 10), indicating that the anchorage-independent growth caused by GM3S was not associated with the PI3K/Akt pathway. Extra pathway(s) may exist to compensate for the PI3K/Akt pathway with respect to anchorage-independent survival of breast tumor cells.

## Discussion

Breast cancer is the most common malignant disease of women. In women with breast cancer, it is not the primary tumor but its metastasis to distant sites that is the ultimate cause of death. Improving our understanding of the molecular mechanisms that underlie the metastatic process might improve clinical management of the disease [29].

It has been demonstrated that levels of total gangliosides and GM3 (N-glycolylneuraminic acid) in breast tumor tissue were significantly higher than in normal tissues [18,24], and ganglioside content was fourfold higher in the invasive human breast cancer cells MDA-MB-231 than in noninvasive MCF-7 cells [29]. In the present study, the results of flow cytometry analysis indicated that GM3 and GD3 gangliosides were significantly higher in the highly metastatic 4T1 cells than in the nonmetastatic 67NR cells. These studies suggested that gangliosides might play roles in breast cancer formation and metastasis.

Metastasis is a rather complex process that occurs through a series of steps that include invasion, intravasation, transport through the circulatory system, arrest at a secondary site, and the extravasation and growth in a secondary organ [30]. The findings presented here suggest that GM3S expression is a multistep modulator of breast cancer cell metastasis. First, gain in motility and invasiveness is essential for most steps in metastasis (the initial step, intravasation, and extravasation). Our findings indicate that GM3S expression played important roles in these steps by affecting ability to migrate/invade. In addition, anchorage-independent growth is thought to be among the fundamental properties of malignant cells [31,32]. In carcinomas, invasion, intravasation, and extravasation are either deprived of matrix or exposed to foreign matrix components. All of these events normally trigger apoptotic processes [30]. In the present study we demonstrated that GM3S significantly enhanced anchorage-independent growth of breast tumor cells. Based on these findings, we hypothesized that GM3S expression could affect almost all steps in breast cancer metastasis.

Some other studies of the roles played by gangliosides in cancer progression support our hypothesis. GD3 and GD2 gangliosides are upregulated in small cell lung cancer cells, and these gangliosides were able to enhance proliferation and invasion of small cell lung cancer [27]. In melanoma cells, GD3 has also been demonstrated to promote proliferation and invasion [3]. In many types of tumor cells (for example, erythroleukemia, lymphoma, neuroblastoma, melanoma, glioblastoma, and renal cell carcinoma cells), gangliosides may be shed into the tumor cell microenvironment and there inhibit antitumor immune responses [33-36]. It has also been demonstrated that gangliosides shed from tumor cells can induce cell migration and *in vivo *angiogenesis [37-40]. Ganglioside levels in sera from breast cancer patients were also significantly higher than in sera from healthy individuals [17]; this implies that shed breast cancer gangliosides play a role in accelerating tumor progression.

Although gangliosides affect cell migration and invasion in some types of tumor cells, only a few investigations have been conducted that focused on the molecular mechanisms involved. Hamamura and coworkers [3] demonstrated that GD3 promoted melanoma cell invasion through activating p130Cas and paxillin; however, invasion of the human keratinocyte-derived SCC12 cell line was suppressed by GM3 ganglioside via inhibition of matrix metalloproteinase-9 activation, disrupting its association with integrin [41]. Some gangliosides, such as GM3 and GM2, inhibited cell motility facilitated by the tetraspanins CD9 and CD82 in some tumor cell lines (for example, colorectal, haptotactic, and bladder cancer cells) [42-45].

In this study we found that a novel mechanism (silencing of GM3S) inhibited migration and invasion of breast cancer cells through activation of the PI3K/Akt pathway. It is generally accepted that the PI3K/Akt axis promotes tumorigenesis by enhancing the survival capacity of cancer cells [46]. However, some recently published evidence indicates that Akt can inhibit breast cancer cell migration and invasion [47]. Previous reports have well demonstrated that GM3 gangliosides can inhibit Akt signaling, and this inhibition occurs mainly through three processes. First, many studies have demonstrated that GM3 gangliosides inhibit phosphorylation of the epidermal growth factor receptor and result in inhibition of PI3K/Akt signaling in varied cell types [48-51]. Second, GM3 and some other gangliosides interact with integrin and consequently inhibit the integrin/integrin-linked kinase/Akt signaling pathway [52-54]. Finally, GM3 treatment markedly increases PTEN expression, which results in inhibition of Akt signaling in colon cancer cells [55]. Here, we demonstrated that GM3S silencing markedly suppressed PTEN expression and subsequently activated the PI3K/Akt pathway in 4T1 cells. Furthermore, the PI3K specific inhibitor LY294002 partially restored the cell migration/invasion ability inhibited by GM3S knockdown in 4T1 cells. This finding clearly indicates that GM3S-associated breast cancer migration and invasion occurred, at least partly, through the PI3K/Akt pathway.

Previous studies revealed that the NFAT transcription factor promoted invasion of breast cancer cells [56,57]. In this report we demonstrated that GM3S silencing suppressed NFAT1 expression and that inhibition of the PI3K/Akt pathway significantly enhanced expression of NFAT1. These findings suggest that inhibition of NFAT1 expression via the activation of PI3K/Akt signaling plays a causal role in the suppression of migration and invasion that is induced by GM3S knockdown in murine breast cancer cells.

## Conclusion

This study demonstrates that GM3S silencing can suppress cell migration, invasion, anchorage-independent growth, and lung metastasis in murine breast cancer cells. The molecular mechanism underlying the GM3S-mediated migration and invasion was found to be inhibition of the PI3K/Akt pathway. To our knowledge, this report is the first to elucidate the involvement of GM3S and mechanisms by which it influences the malignant properties of breast cancer cells.

## Abbreviations

CMP = cytidine 5'-monophosphate; DME-10 = Dulbecco's modified Eagles medium supplemented with 10% fetal calf serum; EGFP = enhanced green fluorescent protein; GAPDH = glyceraldehyde-3-phosphate dehydrogenase; GM3S = CMP-N-acetylneuraminic acid:lactosylceramide 2,3-sialyltransferase; NFAT = nuclear factor of activated T cell; PI3K = phosphoinositide-3 kinase; PTEN = phosphatase and tensin homolog; RT-PCR = reverse transcription polymerase chain reaction; siRNA = small interfering RNA.

## Competing interests

The authors declare that they have no competing interests.

## Authors' contributions

YG and JZ participated in designing the study, conducted cell line transfection, immunoblotting analysis and animal experiments, and drafted the manuscript. WM conducted the flow cytometry analysis and immunoblotting analysis. JY participated in vector construction. FH and XL participated in the design of the study and conducted the statistical analysis. WY conceived of the study, participated in its design and coordination, and helped to draft the manuscript. All authors read and approved the final manuscript.
